# Liver, Tumor and Viral Hepatitis: Key Players in the Complex Balance Between Tolerance and Immune Activation

**DOI:** 10.3389/fimmu.2020.00552

**Published:** 2020-03-27

**Authors:** Matti Sällberg, Anna Pasetto

**Affiliations:** Division of Clinical Microbiology, Department of Laboratory Medicine, Karolinska Institutet, Stockholm, Sweden

**Keywords:** liver, macrophage, T cell, T cell therapy, cancer

## Abstract

Liver cancer is the third most common cause of cancer related death in the World. From an epidemiological point of view the risk factors associated to primary liver cancer are mainly viral hepatitis infection and alcohol consumption. Even though there is a clear correlation between liver inflammation, cirrhosis and cancer, other emerging liver diseases (like fatty liver) could also lead to liver cancer. Moreover, the liver is the major site of metastasis from colon, breast, ovarian and other cancers. In this review we will address the peculiar status of the liver as organ that has to balance between tolerance and immune activation. We will focus on macrophages and other key cellular components of the liver microenvironment that play a central role during tumor progression. We will also discuss how current and future therapies may affect the balance toward immune activation.

## Introduction

The liver is a multi-tasking organ responsible for many crucial functions in the body. It is mostly known for its metabolic and detox work, in fact, it participates in the metabolism of fats (making bile), it stores and releases glucose and it clears harmful substances from the blood. The liver receives about 1.5 L of blood every minute coming from the digestive tract and its job is also to prevent and fight infections coming from the bloodstream. With such massive amount of blood, it is easy to imagine the countless potential antigens that the liver encounters at any given time. To prevent unnecessary inflammation and autoimmune disease the liver harbors different cellular components that work together with complex loops of interactions to maintain the organism homeostasis (1). Immune tolerance is therefore needed in the liver as safe measure to prevent tissue damage that would compromise the metabolic functions of this organ (2). The down side of this protective effect is that some infections, like viruses targeting hepatocytes (3) and even cancer have the possibility to escape the immune-response and give rise to potentially lethal liver diseases (4–7). A general view is that tolerance can be induced by both an insufficient/tolerogenic priming of effector T cells (8, 9) and directly through a regulatory T-cell response (10, 11). Kupffer cells, the specific tissue-resident macrophages of the liver, have a key role in promoting tolerance (12). This review will examine the major cellular components of the liver and tumor microenvironment, their role in controlling the balance between tolerance and activation and the potential therapeutic interventions to tilt the balance against liver cancer progression.

## Antigen Presentation in the Liver

### Role of Kupffer Cells

**FIGURE 1:**
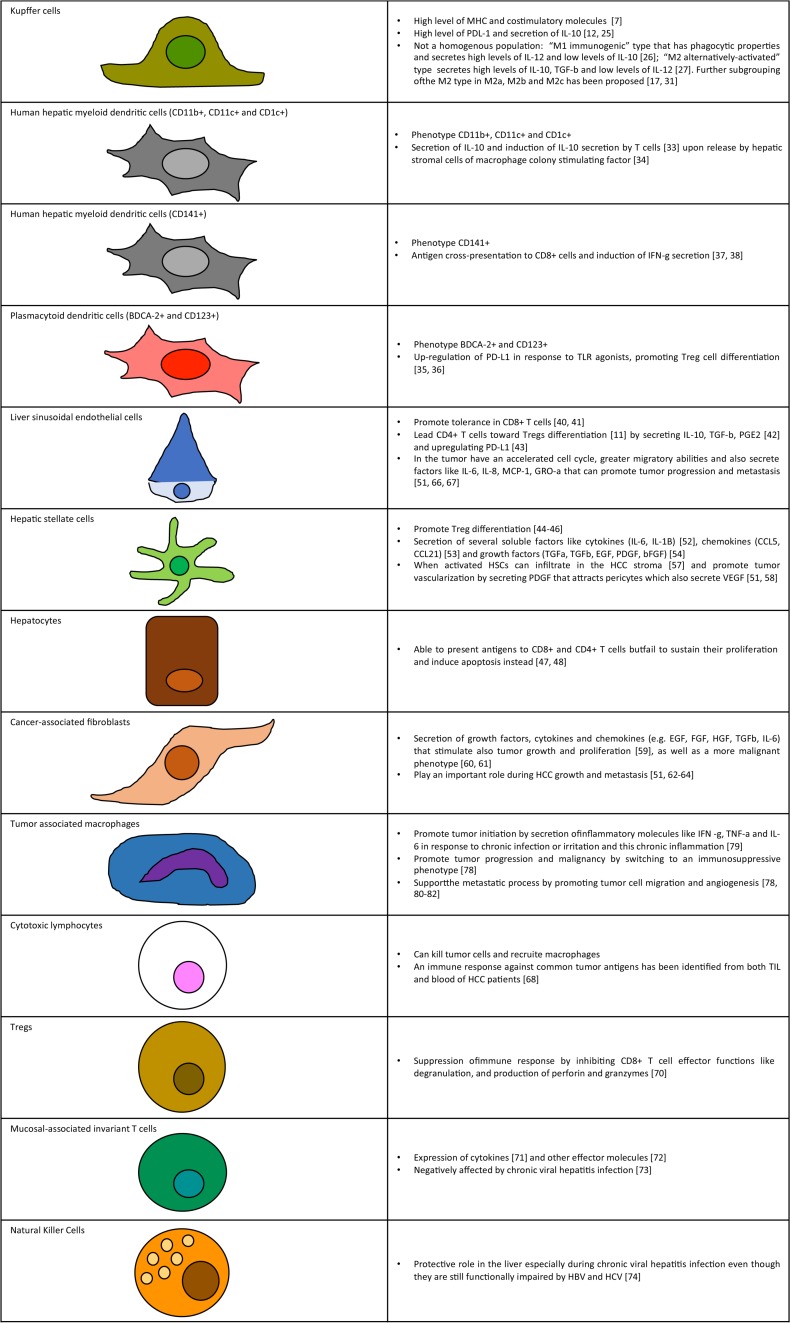
Key cellular components of liver tumor microenvironment. Schematic representation of cell types and summary of functions in tumor microenvironment.

**FIGURE 2:**
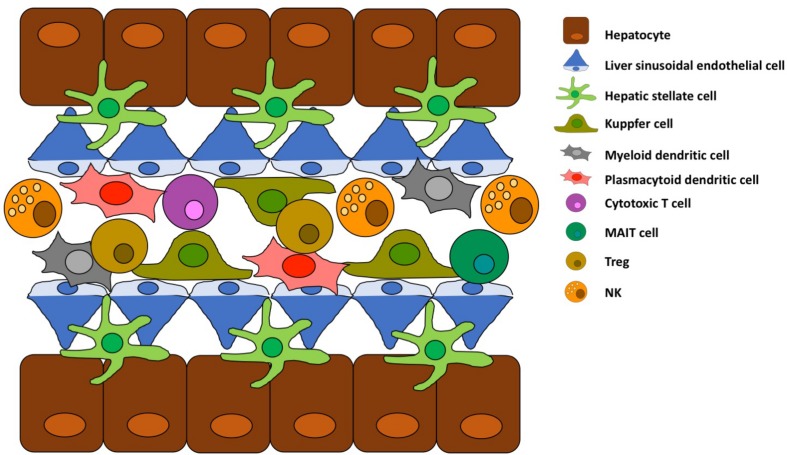
Schematic structure of liver sinusoid. Key cell types and their spatial location are schematically represented.

Kupffer cells (KCs) are liver-resident macrophages, they originate from fetal liver-derived erythromyeloid progenitors and their population is maintained by self-renewal and not by infiltrating monocytes (Figure 1; 13, 14). KCs are located in the hepatic sinusoid as represented in Figure 2, where pathogens enter the liver via portal or arterial circulation (15). The KC population represent a first line defense against infectious particles and potentially immune reactive particles entering the blood stream from the gut (16, 17). KCs express several scavenger receptors (18) such as Toll-like, complement, antibody receptors which allow them to activate danger-associated molecular patterns and internalize, and kill pathogens (15, 19). They also contribute to the initiation of an innate immune response by secretion of cytokines and chemokines (20). The KCs have a potent phagocytic activity not only for the above-mentioned blood-born pathogens but also for other particles/complexes/debris originating from dead erythrocytes and cells of the hepatic parenchyma. Other functions of KCs are related to the iron (21), bilirubin (22) and cholesterol metabolism (23).

The role of KCs in antigen presentation has been considered to be limited based on experiments where isolated mouse KCs only induced a low T-cell proliferation against soluble antigens *in vitro* (24). Other experimental *in vivo* set-ups (12) showed that KC were able not only to phagocytize particle-bound antigens but also subsequently induce a tolerogenic T-cell response against those antigens. This could be measured by induction of local T-cell proliferation, expansion of Foxp3 + IL-10 + OTII T regs *in vivo*. A high level expression of PDL-1 and secretion of IL-10 by KCs strongly suggests that this cell type can present antigens to CD4+ T cells but then with the goal of inducing tolerance (12, 25).

It is important in this regard to keep in mind that KCs are not a homogenous population. As with other macrophage-like cells, two distinct subsets have been identified, one “M1 immunogenic” type that has phagocytic properties and secretes high levels of IL-12 and low levels of IL-10 (26) and another “M2 alternatively activated” type that secretes high levels of IL-10, TGF-b and low levels of IL-12 (27). When TLRs are engaged the KCs usually act as M2 type (28). When it comes to antigen presentation, several evidences implicate KCs as capable antigen presenting cells (APCs) but due to their up-regulation of inhibitory molecules like PD-L1 (29) and also Fas ligand (30) their major role is likely to be suppression T cell activation and induction of Tregs (10). The classification in M1 and M2 types is not exhaustive and does not reflect the complexity and diversity of functions among this cell population. Further subgrouping of the M2 type in M2a, M2b, and M2c has indeed been proposed. These subgroups express different markers and respond to different stimuli (17, 31). It is important to note that tumor-associated macrophages, which will be discussed later, have a M2 phenotype but a distinct transcriptional profile that promotes tumor angiogenesis (17, 32).

### Antigen Presentation by Other Cell Types

**FIGURE 3:**
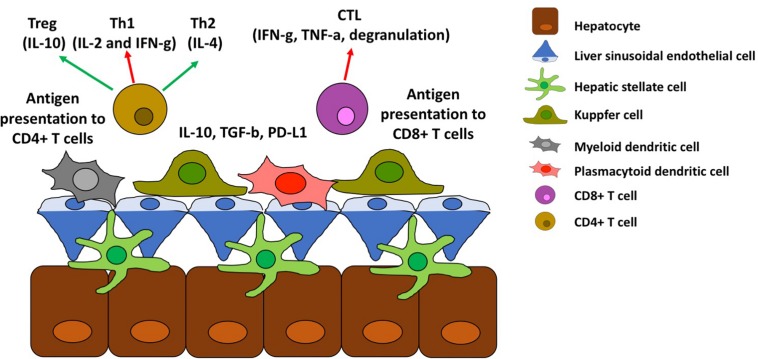
Antigen presentation in the liver. Schematic representation of antigen presentation to CD4+ and CD8+ T cells and its effect toward tolerance (red arrows) or immune activation (green arrow).

A schematic representation of antigen presentation to CD4+ and CD8+ T cells is depicted in Figure 3. Kupffer cells are not the only cell type in the liver that function as APC but inducing tolerance instead of immune activation. Human hepatic myeloid dendritic cells (DC) (phenotypically characterized as CD11b+, CD11c+, and CD1c+) secrete IL-10 and induce IL-10 secretion by T cells (33) upon release by hepatic stromal cells of macrophage colony stimulating factor (34). Plasmacytoid DC (phenotypically characterized by expression of BDCA-2 and CD123) are also present in the liver and up-regulate PD-L1 in response to TLR agonists, promoting Treg cell differentiation (35, 36). Even though the liver APCs are skewed toward inducing tolerance there is also a population of myeloid DC (CD141+) that is able to cross-present antigen to CD8+ cells and induce IFN-g secretion (37, 38).

Other liver parenchymal cells also express MHC and costimulatory molecules and are capable of antigen presentation, although most of them still induce tolerance against the antigens presented. Endothelial cells, located at the liver sinusoids and termed liver sinusoidal endothelial cells (LSECs), promote tolerance in CD8+ T cells (39, 40) and lead CD4+ T cells toward Tregs differentiation (11) by secreting IL-10, TGF-b, PGE2 (41) and upregulating PD-L1 (42).

Hepatic stellate cells (HSCs) reside along the sinusoids and regulate the blood flow through these veins. HSCs can present antigens to T cells but again to promote Treg differentiation (43–45).

Lastly hepatocytes seem to be able to present antigens to CD8+ and CD4+ T cells but fail to sustain their proliferation and induce apoptosis instead (46, 47).

### Viral Hepatitis Infection: Chronic Inflammation and Liver Cancer

The three major viruses responsible for viral hepatitis are the hepatitis B virus (HBV), the hepatitis C virus (HCV), and the hepatitis D virus (HDV), each estimated to be chronically infect 257 milion, 140 milion, and 15 milion people worldwide, respectively (48). While the viruses are quite different as HBV is a double stranded-DNA virus belonging to the Hepadnaviridae family, HCV is a single-stranded + RNA virus belonging to the Flaviviridae family, and HDV a single stranded circular -RNA viroid, they share common pathological paths. All viruses infect hepatocytes but are not directly cytolytic, the cell damage in fact is due to the anti-viral immune response, such as direct killing by cytotoxic CD8+ T cells or NK cells (49) or the action of inflammatory cytokines (50). During immune activation, liver resident APCs can activate virus-specific T cells which are then responsible for recognition and killing of infected hepatocytes and secretion of inflammatory cytokines (50). Acute hepatitis is associated with viral clearance in 95% of HBV infections in adults but only 5% of vertical infections, and in about (49). 25% of HCV infections For HDV, a satellite virus to HBV, acute infections occur when HBV and HDV coinfect a host (clearance rate is 95%), whereas most become chronically infected by HDV when appearing as a superinfection of HBV carriers. When viral clearance is not achieved and the virus is able to persist in the host, this is associated with a chronic inflammation of the liver that can lead to fibrosis, cirrhosis and also cancer. Thus, it is most likely that the persisting hepatic inflammation is the key driver for transformation of hepatocytes to cancer cells. The mechanisms of persistence differs for the different viruses, although a common strategy is to disturb and impair the host immune response. For HBV T cell tolerance, or dysfunction, is a key mechanism of persistence (51). In neonatal vertical infection, the high rate of chronicity is most likely explained by the immature immune response of the host in combination with overproduction of some HBV proteins. In particular one of these proteins is able to pass the placenta and thereby, when presented during schooling of the immunesystem, is perceived as self proteins (52, 53). Hence, these T cell clones are deleted. Other mechanisms of persistence used by HBV, are the constant presence of viral antigens causing immunactivation and then anergy or dysfunction. Finally, the over production and secretion of HBV surface antigen (HBsAg) particles mainly composed of small HBsAg, effectively blocks neutralizing anti-HBs antibodies. Thus, viral particles whose surface is mainly composed of PreS1 and PreS2 can escape these antibodies and infect new cells. Taken together, these factors most likely explain why HBV is able to persist, despite being a genetically stable virus. In contrast, several observations point to the fact that the higher rate of chronic infections due to HCV as compared to HBV is associated with the better ability of HCV to evade adaptive immunity (54–56), probably due to its extreme mutation rate (57). Thus, HCV persists as a virus constantly changing to evade both B and T cells. For HDV the mechanisms of persistence are less well known, except that HDV accelerates the tissue damage and disease during dual infection. Chronic hepatitis, regardless of the virus causing it, is characterized by a persistent presence of the viral antigens, which cause a continuous T cell stimulation. This chronic stimulation is regulated by immune checkpoint (i.e., expression of PD-1) that limit the effector functions of the virus-specific T cells resulting in a loop of low-grade inflammation and ineffective viral clearance (58, 59). Another important actor in the balance between immune activation and suppression are Tregs. Effective viral clearance is achieved through cytotoxicity that can be very dangerous if uncontrolled, or too massive. An excessive tissue damage needs to be prevented for the sake of host survival, and therefore the role of Tregs can be seen as a safe measure against fatal liver injury. Mouse studies modeling acute HBV infection observed an increased liver damage after Treg depletion (60), also HCV mouse models showed a transient peak of Tregs during acute infection, and a persistent increase in Tregs frequency in chronic infection (61). It is not yet clear wheather Tregs expansion is a cause or a consequence of chronic infection, but certainly this phenomenon is highlighting the complex interplay and immunological balance occurring in the liver during viral infection. The major long-term consequence of chronic infection is the occurrence of fibrosis and cirrhosis that impair liver functionality and also the insurgence of cancer. Treg expansion has been observed in samples of hepatocellular carcinomas (HCCs) and cirrhotic tissue (62). In this study (62), the expression of OX40 (a activation marker) correlates with intratumoral Treg frequency and other markers of proliferation like Ki67; OX40L on the other hand is expressed on myeloid liver infiltrating cells that co-localize with Tregs and directly correlates with HCV viral load. So this is an example of interplay between the host and the virus that is trying to escape the immune system by inducing Tregs via OX40L-OX40 interaction (63, 64).

## Primary Tumors in the Liver (Hcc)

Hepatocellular carcinoma is one of the leading causes for cancer-related death in the world. HCC is mostly asymptomatic and the diagnosis is usually made at a late stage, whereby the prognosis generally is poor. Several risk factors have been identified for HCC, such as chronic viral hepatitis, excessive alcohol consumption, obesity, fatty liver, and diabetes (65). Although the presence of these risk factors aid in monitoring specific patient populations, the molecular mechanism(s) behind tumor initiation, progression, and metastasis are poorly understood and this poses a limitation to find efficient therapeutic approaches.

Hepatocellular carcinoma is associated with the dysregulated growth of hepatocytes that form dysplastic nodules resulting in chronic liver damage or cirrhosis. Mouse models have shown that the hepatocytes in this context have a higher expression of vimentin and type I collagen, suggesting that they are acquiring mesenchymal phenotype (66).

Although the specific mechanism has not been clarified, increasing evidence is pointing at the role of tumor microenvironment in HCC pathogenesis (67). In the following sections we will describe the main cellular components of the liver tumor microenvironment considering the primary tumor but also the metastatic deposits originated from other common tumor types like colon and breast cancer.

## Main Cellular Components of the Tumor Microenvironment

### Hepatic Stellate Cells (HSCs)

A summary of cellular components of the tumor microenvironment is represented in Figure 1. These cells are part of the liver connective tissue and are located on the perisinusoidal space. HSCs have many homeostatic functions such as vitamin A accumulation, synthesis of collagen, expression of several soluble factors like cytokines (IL-6, IL-1B) (68), chemokines (CCL5, CCL21) (69), and growth factors (TGFa, TGFb, EGF, PDGF, and bFGF) (70). Liver damage can cause proliferation of HSCs and cytoskeleton remodeling (71, 72). When activated HSCs can infiltrate in the HCC stroma (73) and promote tumor vascularization by secreting PDGF that attracts pericytes which also secrete VEGF (67, 74).

### Cancer-Associated Fibroblasts (CAFs)

This type of fibroblasts mediates several interactions between the tumor and the liver stroma. They secrete growth factors, cytokines and chemokines (e.g., EGF, FGF, HGF, TGFb, and IL-6) that stimulate also tumor growth and proliferation (75), as well as a more malignant phenotype (76, 77). Several evidences showed that CAFs play an important role during HCC growth and metastasis (67, 78–80).

### Endothelial Cells

The main role of these cells is to promote tissue vascularization. The morphology of blood vessels is different in tumors compared to normal tissues, probably due to increased permeability, and this seems to be related to molecular and functional differences of epithelial cells (81). In particular, the endothelial cells present in the tumor have an accelerated cell cycle, greater migratory abilities and also secrete factors like IL-6, IL-8, MCP-1, and GRO-a that can promote tumor progression and metastasis (67, 82, 83).

### Immune Cells

#### Tumor Infiltrating Lymphocytes (TILs)

Tumor Infiltrating Lymphocytes can play an important role for tumor progression, like Tregs, but can also be the source of powerful anti-tumor cells that can be used for therapy. An immune response against common tumor antigens has indeed been identified from both TIL and blood of HCC patients (84). The presence of Tregs in tumor is usually associated with a worse prognosis (85) since this cell type suppress the immune response by inhibiting CD8+ T cell effector functions like degranulation, and production of perforin and granzymes (86). Recent studies are also highlighting the importance of a mucosal-associated invariant T cell population (MAIT) that is able to activate and express cytokines (87) and other effector molecules (88), MAIT cells seem also to be negatively affected by chronic viral hepatitis infection (89).

Natural killer cells (NK) account for up to 50% of the total number of lymphocytes present in the liver. It has been shown that NK have a protective role in the liver especially during chronic viral hepatitis infection even though they are still functionally impaired by HBV and HCV (90). In HCC, it seems that the NK functionality is also impaired but the use of genetically modified NK cells in combination with kinase inhibitors has provided promising results by *in vivo* models (91, 92).

#### Tumor Associated Macrophages (TAMs)

Macrophages are frequently found in tumors and according to a high number of clinical and experimental data they seem to promote tumor initiation, progression and even metastasis (93, 94). The mechanism behind the contribution of macrophages to cancer initiation and progression seems to be caused at two major steps: initially macrophages secrete inflammatory molecules like IFN-g, TNF-a and IL-6 in response to chronic infection or irritation and this chronic inflammation seems to be causal to tumor initiation (95). Once the tumor is established, the macrophages switch to an immunosuppressive phenotype promoting progression and malignancy (94). Moreover, TAMs support the metastatic process by promoting tumor cell migration and angiogenesis (94, 96–98). In HCC a role of infiltrating monocytes and KCs seems to be to drive tumor progression and metastasis (99). The presence of TAMs is also associated with increase tumor burden an higher metastasis rate in both HCC patients and mouse models of liver cancer (100). In particular the ability of macrophages to secrete cytokines, chemokines and growth factors seems to be a crucial component of tumor initiation and proliferation (99, 101). KCs have a pro-inflammatory function that seems to be particularly important for HCC initiation (102). At later stages when the tumor is already established their ability to express PD-L1 and secrete immunosuppressive cytokines like IL-10 further contribute to promote tumor progression due to inhibition of effector lymphocytes like CD8+ T cells (14, 103, 104).

### Microenvironment of Metastatic Deposits in the Liver

As already mentioned the liver receives a massive amount of blood which may help to explain the transport and hepatic entry of metastatic tumor cells coming from other organs of the body. The liver is indeed the main site of metastasis for several types of cancer, like colon, pancreas, melanoma, breast cancer, and sarcomas (105). The same cellular components that play an important role for tumor initiation and progression of primary liver cancer are also likely to be involved in facilitating the establishment of metastasis (106, 107). Another cell type that has been recently shown to be an important player for development of metastasis are marrow-derived immune cells recruited to the liver. A mouse model of metastatic pancreatic ductal adenocarcinoma revealed an interplay between the different cellular types in the liver that ultimately favored the metastatic deposit formation (108). In this model, tumor cells secrete exosomes that are taken up by KCs that then increase TGFb production affecting HSC that also increase fibronectin production with ultimate recruitment of bone marrow-derived macrophages. Another study found that exosomes from pancreatic cell lines able to metastasize in the liver, contain integrins that can fuse preferentially with KCs. These data also suggest that exosomes may have a role in disease progression and specific organ metastasis depending on the integrins that they contain (109). In general as a metastatic circulating cell survive the first line of defense offered by KCs and HSC (110), the liver milieu, rich of growth factors, proinflammatory molecule such as S100A8, and immune-suppressive cytokines, favors the formation of pre-metastatic niches (109, 111–114). In mouse models, NK cells are shown to play an important role in immune surveillance and prevention or delay of metastatic formation in the liver (115–117). Cancer cells are able to escape direct NK cytotoxicity by forming clusters with other cancer cells (118).

## Targets for Therapy: Can We Tilt the Balance?

As described above the level of complexity of the liver and liver-cancer microenvironment is very high. Due to the multiple cellular components, cytokines, chemokines, and physical disposition around the liver sinusoid, it is unlikely that one single approach could be able to break the vicious loop of inflammation, growth stimulation, and immune-tolerance that characterize the interplay between liver and cancer. Despite this, we believe that a combined approach of several therapies targeting different components of the tumor microenvironment may be effective. In the following sessions the available and potential future therapies for liver cancer are reviewed.

### Target Microenvironment With Drugs

It is possible to specifically block the signaling pathways used by cancer cells to take advantage of the stroma cells and their growth stimulation (119). For HCC the two most studied pathways are the ones that promotes inflammation and angiogenesis (120). The only approved drug for advanced HCC is Sorafenib, a multi-kines inhibitor that target VEGFR, Raf-kinase and PDGFR, but unfortunately its efficacy is limited (121). Other drugs with similar mechanism of action are currently in experimental evaluation (122, 123).

### Target Microenvironment With Physical Agents

One curative option for early stage HCC is ablative surgery. If the tumor is removed completely or the liver is transplanted there is a chance that the tumor will not come back. Most of patients are unfortunately diagnosed at a very late stage when surgical alternatives are not a possibility anymore (65). Common ablative procedures like radiation, cryoablation, and trans arterial chemoembolization (TACE) are also used at a late stage as palliative treatment. Physically destroying tumor cells may have the advantage to release tumor-antigens that can be taken-up by APCs and be cross-presented to T cells. Increased tumor-specific cytotoxicity has indeed been detected after this type of therapies (124–126). The potential side effect of this treatment is that the presence of PD-L1 on most of APCs in the liver could actually inhibit the tumor-specific T cell response (127) so one obvious measure would be to combine ablation therapy with PD-1 blockade treatment (128, 129). This is currently tested in various clinical trials.

### Target Microenvironment With Immunotherapy

Immune suppression and tolerance are key factors in the induction, progression and metastasis of cancer in the liver. It is logical to assume that targeting the immune cells in the liver, reverting what cause their anergic state, may offer a solution for cancer treatment. Several immunotherapy trials have been conducted for HCC with cell therapy (LAK and TIL), cytokines and check-point inhibitors, dendritic cells vaccines and combinations of the above (129). The outcomes of these trials are promising but still not enough to offer a complete remission for HCC. Possibly, the particular microenvironment of the liver presents more challenges compared to other epithelial cancers that were successfully treated by adoptive cell therapy (130–134).

One current promising immunotherapeutic approach is the use of check-point inhibitors that target PD-1 and CTLA-4 promoting T cell activation (135). Recent clinical trials with the anti-PD-1 monoclonal antibodies Nivolumab showed promising anti-tumor activity in some patients with advanced HCC (136).

## Conclusion

In this review we focused on the interplay within the liver between non-parechymal phagocytig and APCs, viruses, and liver cancer microenvironments. This highlights the complexity and intricate interplay of different factors. Due to the huge number of antigens going through the liver, the physiological need of having a “safe-guard” mechanism to prevent harmful immune-activation and liver damage is very understandable. The draw-back of this safe-guard mechanism is that pathogens like viruses or cancer, both primary and metastatic tumor cells, may establish and proliferate. We discussed some of the potential targets in the tumor microenvironment that can promote activation for the immune-system and potentially reverse the tolerance against cancer antigens. The milieu in the liver is highly complex and it is probably unrealistic to think that just a single “magic bullet” therapy could tilt the balance an mediate cure. It is most likely necessary to attack many or all the key players to effectively intervene with tumor progression. A combination of physical ablation, immunotherapy and other molecular drugs may indeed be needed.

One of the key components in the tumor microenvironments are TAMs. The biology of macrophages in general is very complex and to some extent still unknown. There are several evidences that suggest a different role of macrophage subgroups depending on their origin (e.g., fetal liver, bone marrow, etc.) and their location in the organism. What is clear is that they can have multiple, and sometimes opposite functions, that can influence clearance or disease progression. More research is needed to better understand the extent of their influence on tumor formation and progression.

## Author Contributions

All authors contributed to the conception and the writing of the review.

## Conflict of Interest

The authors declare that the research was conducted in the absence of any commercial or financial relationships that could be construed as a potential conflict of interest.
